# Living Donor Re-transplantation for Repeated Acute Liver Failure

**Published:** 2018-02-01

**Authors:** V. Ince, C. Kayaalp, E. Otan, F. Ozdemir, A. Dirican, H. I. Toprak, C. Aydin, C. Ara, S. Yilmaz

**Affiliations:** 1 Inonu University, Liver Transplantation Institute, Department of General Surgery, Turkey; 2 Inonu University, Liver Transplantation Institute, Department of Anesthesiology, Turkey

**Keywords:** Liver failure, acute, Living donors, Transplantation

## Abstract

Emergency liver transplantation (LT) for acute liver failure (ALF) is a life-saving treatment. Occurrence of this situation in the same patient twice is very rare. Herein, we describe a patient who underwent two emergency LTs for ALF, both from living donors. When she was 26 years old, she underwent a right lobe living donor LT (LDLT) from her sister for ALF due to use of herbal weight loss medications. The next 3 years were uneventful but another ALF developed during a terminal stage pregnancy (37^th^ week). Despite medical treatment, her liver functions worsened, and the baby was delivered by caesarean section. The second time, her brother was the donor and she recovered after the emergency right lobe re-LDLT. Both patient and baby were well at the 2-month follow-up. As far as we know, there is no reported similar case, and we concluded that LDLT is a paramount treatment option for both primary and secondary ALFs.

## INTRODUCTION

Acute liver failure (ALF) is a clinically serious condition with a high risk of mortality. Etiologies vary from pediatric to adult patients and from developing countries to industrial societies [1]. Emergency liver transplantation (LT) is a unique treatment for those in the irreversible phase of liver failure, regardless of its etiology and patient age. Under this circumstance, living donor LT (LDLT) presents an advantage to shorten the organ waiting period compared with that of cadaveric LT [2]. Although drug-related ALF has been reported among patients who underwent LT, no record of repeated ALF in a patient with a previous history of LT due to ALF exists in the English medical literature [3]. Herein, we initially present a patient with a previous history of LDLT due to ALF who underwent a second LDLT due to repeated ALF.

## CASE REPORT

A 29-year-old woman underwent LDLT due to ALF induced by consumption of herbal tea. The donor was her brother. The post-operative period was uneventful, and the patient became pregnant after getting married. The early course of the pregnancy was stable with single tacrolimus immunosuppressive treatment (blood level of 2.5–10.5 ng/mL). However, her serum transaminase levels increased during the gestational week 37. She underwent a caesarian section and delivered a healthy boy weighing 2900 g. She was admitted to the LT ward post-operatively. Her physical examinations were normal in a conscious state. She had a platelet count of 207×10^3^/mm^3^, international normalized ratio of 2.0, aspartate transaminase of 1441 U/L, alanine transaminase of 1351 U/L, total bilirubin of 11.6 mg/dL, ammonia level of 224 (normal range: 0–109) µg/dL, and serum lactate level of 20 (normal range: 4.5–19.8) mmol/L. Doppler ultrasonography and multislice abdominal computed tomography revealed normal anatomical and vascular structures. Magnetic resonance cholangiography was also normal. The blood tacrolimus level was 9.7 ng/mL; no clinical or laboratory signs of infection were apparent. She was admitted to the intensive care unit (ICU) following stage I-II hepatic encephalopathy that developed and progressed to stage III. Her emergency was reported to the National Organ Sharing Association for Cadaveric Liver Procurement. Her brother was evaluated as a donor. She was treated with two sessions of a molecular adsorbent recirculating system and three sessions of plasmapheresis as a bridging procedure to LT. No significant improvement was observed. She developed tonic-clonic contractions resembling a seizure and underwent LDLT in which her brother was the donor. During the intra-operative exploration, adherent tissues were removed and the graft was observed to be pale and stiff on palpation. Following removal of the transplanted graft, retransplantation with the new right-lobe liver graft was performed successfully (no blood products were administered intra-operatively). The patient was taken to the ICU and extubated on post-operative day 1. She was transferred to the liver transplant patients’ ward on post-operative day four and was discharged on postoperative day 19 following an uneventful course. Zone 3 necrosis and inflammation was diagnosed by pathological examination of the removed graft. The patient and her baby were living uneventfully two months post-operatively.

## DISCUSSION

In Turkey, the most common etiologies for ALF among the adult population are viral (40%) (mainly hepatitis B virus), drug toxicity (20%), and intoxication (11%). The survival rate of patients treated with LT is significantly higher than that of patients treated conservatively (65% vs. 36%, p<0.001) [1, 4-6]. The timing for LT in a patient with ALF remains controversial. King’s College criteria are still used for this purpose. However, brain death is common among these patients following LT and patients are lost with a functioning liver graft [7]. Following progression of encephalopathy to grade III, our emergency case was reported to the National Organ Sharing Association for Cadaveric Liver Procurement. However, seizures led to the decision of a re-LDLT, which minimized time loss and provided a definitive treatment for the rapidly progressing condition. Similarly, this patient’s state of emergency was declared before her initial LT. However, LDLT was performed for lack of a suitable cadaveric graft. LDLT reduces the waiting period for the graft and improves survival.

Several drug-related liver failures following LT cases have been reported, most of which were resolved with medical treatment but one case underwent re-transplantation [3, 8]. All of these cases had an initial diagnosis other than ALF, which makes our case unique.

ALF during the course of pregnancy is not rare. Fatty liver disease in pregnancy, viral hepatitis (especially, hepatitis E virus), and HELLP syndrome (Hemolysis, Elevated Liver Enzymes, and Low Platelet Count) are well-known conditions related to ALF. None of these conditions in which pre-eclampsia co-exists were diagnosed in our case. After rejection was clinically ruled out, conservative treatment was initiated, but it did not resolve the condition and led to an emergency report to the National Organ Sharing Association. In a study of 18 cases from our center presenting risks and results of pregnancy after LT, no pregnancy-related liver failure was observed. The rate of natural childbirth was 65.4% and the most frequent maternal complication was hypertension with a rate of 16.6% [9]. No similar study reporting on pregnancy following LT has reported ALF.

As a result, no other person in the world is so unlucky and so lucky. A patient with ALF may be treated with LDLT. However, undergoing a second LDLT due for lack of a cadaveric graft is an extremely rare condition. Our case is the first individual report of this condition.
